# National Latinx AIDS Awareness Day — October 15, 2019

**DOI:** 10.15585/mmwr.mm6840a1

**Published:** 2019-10-11

National Latinx AIDS Awareness Day, October 15, is observed each year to focus on the continuing and disproportionate impact of human immunodeficiency virus (HIV) infection and acquired immunodeficiency syndrome (AIDS) on Hispanics/Latinos in the United States. In 2017, 26% of newly diagnosed HIV infections occurred in Hispanics/Latinos (*1*). Seventy-five percent of these newly diagnosed HIV infections in Hispanics/ Latinos were in men who have sex with men (MSM), and an additional 3% were in MSM who inject drugs (*1*).

An analysis of the behaviors of Hispanic/Latino MSM included in CDC’s National HIV Behavioral Surveillance system found that nearly 75% reported having had condomless anal sex during 2017 (*2*). However, because some of these MSM reported using preexposure prophylaxis (PrEP), fewer than 60% of those who were non–U.S.-born and fewer than 50% of those who were U.S.-born were having unprotected anal sex (*2*).

National Latinx AIDS Awareness Day is an opportunity to encourage increased HIV prevention efforts among Hispanics/Latinos. CDC supports testing, linkage to and engagement in care and treatment, and other efforts to reduce the risk for acquiring or transmitting HIV infection. More information is available at https://www.cdc.gov/hiv/group/racialethnic/hispaniclatinos/index.html and https://www.cdc.gov/hiv/group/msm/hispanic-latino.html.
